# Serum antibody responses to pneumococcal colonization in the first 2 years of life: results from an SE Asian longitudinal cohort study

**DOI:** 10.1111/1469-0691.12286

**Published:** 2013-07-05

**Authors:** P Turner, C Turner, N Green, L Ashton, E Lwe, A Jankhot, N P Day, N J White, F Nosten, D Goldblatt

**Affiliations:** 1 Shoklo Malaria Research UnitMae Sot, Thailand; 2 Mahidol-Oxford Tropical Medicine Research UnitBangkok, Thailand; 3 Centre for Tropical Medicine, University of OxfordOxford, UK; 4 Immunobiology Unit, Institute of Child Health, University College LondonLondon, UK

**Keywords:** Antibody, capsule, colonization, polysaccharide, protein, *Streptococcus pneumoniae*, vaccine

## Abstract

Assessment of antibody responses to pneumococcal colonization in early childhood may aid our understanding of protection and inform vaccine antigen selection. Serum samples were collected from mother-infant pairs during a longitudinal pneumococcal colonization study in Burmese refugees. Maternal and cord sera were collected at birth and infants were bled monthly (1–24 months of age). Nasopharyngeal swabs were taken monthly to detect colonization. Serum IgG titres to 27 pneumococcal protein antigens were measured in 2624 sera and IgG to dominant serotypes (6B, 14, 19F, 19A and 23F) were quantified in 864 infant sera. Antibodies to all protein antigens were detectable in maternal sera. Titres to four proteins (LytB, PcpA, PhtD and PhtE) were significantly higher in mothers colonized by pneumococci at delivery. Maternally-derived antibodies to PiuA and Spr0096 were associated with delayed pneumococcal acquisition in infants in univariate, but not multivariate models. Controlling for infant age and previous homologous serotype exposure, nasopharyngeal acquisition of serotypes 19A, 23F, 14 or 19F was associated significantly with a ≥2-fold antibody response to the homologous capsule (OR 12.84, 7.52, 6.52, 5.33; p <0.05). Acquisition of pneumococcal serotypes in the nasopharynx of infants was not significantly associated with a ≥2-fold rise in antibodies to any of the protein antigens studied. In conclusion, nasopharyngeal colonization in young children resulted in demonstrable serum IgG responses to pneumococcal capsules and surface/virulence proteins. However, the relationship between serum IgG and the prevention of, or response to, pneumococcal nasopharyngeal colonization remains complex. Mechanisms other than serum IgG are likely to have a role but are currently poorly understood.

## Background

Nasopharyngeal colonization is thought to always precede pneumococcal infection 1. Immune responses to colonization are complex: experimental murine models suggest a limited role for antibody and that an IL-17A-mediated CD-4^+^ T-cell pathway is the dominant mechanism involved in immunity to pneumococcal colonization 2,3. However, colonization-related serum immunoglobulin G (IgG) antibody responses to capsular polysaccharide and surface/virulence proteins have been described in adult humans 4,5, although these antibodies may not protect against colonization 6. In children aged <2 years, studies have demonstrated limited serum anti-capsular antibody development in the absence of pneumococcal immunization 7. Several pneumococcal proteins are immunogenic in young children and antibody development is correlated with pneumococcal exposure 8. Most studies in the literature use relatively infrequent sampling of blood to assess development of natural immunity, few combine with nasopharyngeal carriage data, and none to our knowledge have ever sampled monthly for 24 months and then performed combined analyses of anti-capsular and anti-protein antibody responses.

Vaccines containing pneumococcal proteins have provided protection against invasive infections in mice 9. Assessments of the relationship between nasopharyngeal colonization and antigen-specific immune responses in young children are critical for prediction of potential carriage-mediated protein vaccine hyporesponsiveness. This is important because serotype-specific hyporesponsiveness has been previously demonstrated for the currently available conjugate vaccines 10.

This manuscript describes the temporal relationships between nasopharyngeal colonization and serum IgG to pneumococcal capsular polysaccharides and surface/virulence proteins over the first 2 years of life in a population where there is early/sustained pneumococcal carriage. Effects of transplacentally-transferred IgG antibodies on infant pneumococcal colonization are explored.

## Methods

### Study population

Mother-infant pairs were followed from birth for 24 months during a pneumococcal colonization and pneumonia study in a camp for Burmese refugees 11,12. At delivery, nasopharyngeal swab (NPS) and serum specimens were collected from the mother and a serum specimen from the umbilical cord. NPS were taken from mother to infant, plus serum from infants, at monthly intervals (1–24 months of age). Pneumococcal vaccines were not available to the study population.

### Pneumococcal nasopharyngeal colonization

Nasopharyngeal swabs were cultured using the WHO standard protocol to detect pneumococcal colonization 13. Briefly, a Dacron-tipped nasopharyngeal swab was used to sample the nasopharynx and the tip immediately excised into a cryotube containing 1 mL STGG (skim milk, tryptone, glucose, glycerol) transport medium. NPS-STGG specimens were transferred back to the laboratory in a cool box before being promptly frozen at−80°C. Ten microlitres of the thawed and vortexed NPS-STGG specimen were cultured overnight on sheep blood-CNA agar (bioMerieux, Marcy L'Etoile, France) at 36°C and 5% CO_2_. All morphologically distinct alpha-haemolytic colonies were subcultured onto plain sheep blood agar (Clinical Diagnostics, Bangkok, Thailand) and *S. pneumoniae* was confirmed by colonial morphology and susceptibility to optochin (Oxoid, Basingstoke, UK). The bile solubility test was used to confirm isolates with equivocal optochin disc susceptibility and those non-typeable by Omniserum (SSI Diagnostica, Hillerod, Denmark). Pneumococcal isolates were serotyped by latex agglutination using a full panel of pneumococcal antiserum (SSI Diagnostica), with Quellung confirmation of equivocal results 14.

### Antigens and serological methods

Serum IgG antibodies to 27 pneumococcal protein antigens were measured using a direct binding electrochemiluminescence-based multiplex assay (Table1). The assay was based on that described for pneumococcal polysaccharide antigens utilizing MesoScale Discovery (MSD, Rockville, MD, USA) technology 15. Pneumococcal reference serum 007 was used as a standard on each plate and assigned a value of 1000 arbitrary units for each antigen 16. Antibody levels in sera from study participants were expressed as a titre with reference to the amount in 007.

**Table 1:** Protein antigens assessed in the study

Name	Internal ID	Protein details	Provided by
CbpA	PP01	Choline binding protein A, without choline binding domain (CbpA NR1XR2P)	GSK
LytB	PP11	Endo-beta-N-acetylglucosaminidase (LytB-T-PO1)	Sanofi Pasteur
LytC	PP02	Lysozyme (LytC C-ter)	GSK
NanA	PP33	Neuraminidase (NanA)	UAB
PcpA	PP13	Choline binding protein (PcpA DC6842)	Sanofi Pasteur
PcsB-1	PP06	Secreted 45 kDa protein (PcsB, SP2216-1)	Intercell
PcsB-2	PP32	Secreted 45 kDa protein (PcsB, Spr2021)	Novartis
PhtD-1	PP03	Pneumococcal histidine triad D (PhtD)	GSK
PhtD-2	PP14	Pneumococcal histidine triad protein (PhtD DC6857)	Sanofi Pasteur
PhtE	PP10	Truncated histidine triad protein (PhtE-T1 DC6286)	Sanofi Pasteur
PiaA	PP09	Part of iron uptake ABC transporter (PiaA)	PATH
PiuA	PP08	Part of iron uptake ABC transporter (PiuA)	PATH
Ply-1	PP12	Pneumolysin (WtPly DC6968)	Sanofi Pasteur
Ply-2	PP17	Pneumolysin (P10V12/13 Ply)	UAB
PsaA	PP04	Pneumococcal surface adhesin A (PsaA, SP1650)	Intercell
PspA-Fam1	PP16	Pneumococcal surface protein A, family 1 (PspA, P18-01/P18-02)	UAB
PspA-Fam2	PP15	Pneumococcal surface protein A, family 2 (PspA, UAB099 P9V63)	UAB
RrgA-T4	PP22	RrgA pilus subunit, adhesin (RrgA, T4)	Novartis
RrgB-T4	PP18	RrgB pilus subunit, backbone (RrgB, T4)	Novartis
RrgB-6B	PP19	RrgB pilus subunit, backbone (RrgB, 6B)	Novartis
RrgB-23F	PP20	RrgB pilus subunit, backbone (RrgB, 23F)	Novartis
StkP	PP05	Serine threonine kinase protein (StkP, SP1732-3)	Intercell
StrH	PP29	Beta-N-acetylhexosaminidase (StrH, Spr0057)	Novartis
SP0609	PP31	Amino acid ABC transporter, amino acid-binding protein	Intercell
SP2027	PP07	Conserved hypothetical protein (Spr1/SP2027)	Intercell
SP2194	PP30	ATP-dependent Clp protease, ATP-binding subunit	Intercell
Spr0096	PP24	LysM domain-containing protein	Novartis

Serum IgG antibody concentrations to capsular polysaccharides 6B, 14, 19F, 19A and 23F were determined by enzyme-linked immunosorbent assay, after adsorption with 22F polysaccharide and cell-wall polysaccharide 17. The assay limit of detection was 0.15 mg/L; results below this were reported as 0.075 mg/L. Serotypes were selected on the basis of inclusion in the 13-valent conjugate vaccine (PCV13) and frequency of carriage in the cohort 11.

### Serum specimens

For anti-protein antibody analyses, all mother and cord blood specimens were included. Infant specimens were selected for anti-protein antibody analyses to obtain good coverage at each sampling point during the first year of life and to include time-points from the second year of life with the largest specimen numbers. For anti-capsular antibody analyses, specimens from infants with complete 24-month sets of both NPS and serum specimens were selected.

### Statistical analysis

Data were analysed using Stata/IC 12.1 (StataCorp, College Station, TX, USA). Antibody concentrations/titres were log-transformed prior to analyses. Student's *t*-test or ANOVA were used to compare groups, with Bonferroni adjustment for multiple comparisons. Proportions were compared using the chi-squared test. The impact of maternally-derived antibodies on the timing of pneumococcal acquisition in infants was explored by survival analysis.

To assess serum antibody responses in relation to nasopharyngeal pneumococcal acquisitions, a subset of infant data was analysed. Pneumococcal acquisitions were defined as the first appearance of a serotype (including non-typeable pneumococci as a ‘type’) in the nasopharynx or the reappearance of the serotype following its absence from ≥2 consecutive NPS. In cases of multiple serotype colonization, all serotypes were considered in the analyses. For each sampling time-point, ratios of antibody concentrations/titres were calculated by dividing the current specimen concentration/titre by the preceding month's concentration/titre. Assessment of receiver-operating characteristic curves for these ratios vs. acquisitions did not reveal a meaningful response cut-off value. Therefore a 2-fold or greater rise in antibody concentration/titre was arbitrarily used to define a ‘response’. Generalized estimating equations with a logistic link and exchangeable correlation structure were used to determine odd ratios (ORs) for an antibody response at each time-point, controlling for age and pneumococcal acquisitions.

### Ethics

Ethical approval was granted by the Faculty of Tropical Medicine, Mahidol University (MUTM-2009-306) and Oxford University (OXTREC-031-06).

## Results

Serum specimens from 230 mothers and 222 infants were included in these analyses (*n* = 2624; Table S1).

### Maternal anti-protein antibody titres/transplacental transfer

Twenty per cent (46/229) of mothers were colonized by pneumococci at delivery. Every mother had measurable serum IgG antibodies to all proteins studied. Geometric mean antibody titres (GMT) to four proteins were significantly higher in colonized women compared with non-colonized women: LytB (1093.5 vs. 747.9, p 0.0002); PcpA (1264.4 vs. 981.3, p 0.04); PhtD (PhtD-1 (830.3 vs. 613.7, p 0.02); PhtD-2 (758.1 vs. 576.4, p 0.02)); PhtE (789.0 vs. 571.3, p 0.02).

In 183 mother-cord blood pairs, effective transplacental antibody transfer was demonstrated: mean cord:mother serum IgG titre ratios were 0.77–1.08 (data not shown).

### Maternal anti-protein antibody titres/time to infant pneumococcal acquisition

Effects of cord serum antibody titres on timing of first pneumococcal acquisition were explored in 179 infants. Age at first pneumococcal acquisition was 45.5 days (median; inter-quartile range (IQR) 15.5–76.5). All but one infant had been colonized by 11 months.

In univariate Cox proportional hazards models, higher serum IgG antibody titres to PiuA and Spr0096 were associated significantly with delayed pneumococcal acquisition (PiuA, hazard ratio (HR) 0.68, 95% confidence interval (CI) 0.46–0.98, p 0.04; Spr0096, HR 0.73, 95% CI 0.57–0.95, p 0.02). There was no evidence of higher cord blood anti-protein antibody titres being significantly associated with earlier pneumococcal acquisition (Table S2). In a multivariate model including environmental/household factors, cord blood antibody titres to PiuA (HR 0.70, 95% CI 0.45–1.08, p 0.1) or Spr0096 (HR 0.82, 95% CI 0.62–1.09, p 0.2) were no longer associated significantly with timing of pneumococcal acquisition. In this model, maternal smoking (HR 1.49, 95% CI 1.01–2.21, p 0.046) was associated with a significantly earlier acquisition age, with similar trends for maternal pneumococcal colonization (HR 1.49, 95% CI 1.00–2.25, p 0.05) and young children in the household (HR 1.39, 95% CI 0.99–1.96, p 0.06). Infants born in the wet season acquired pneumococci later than those born in the hot or cool seasons (HR 0.62, 95% CI 0.40–0.95, p 0.03) (Table S2).

### Anti-protein antibody kinetics in infants

**fig 1:**
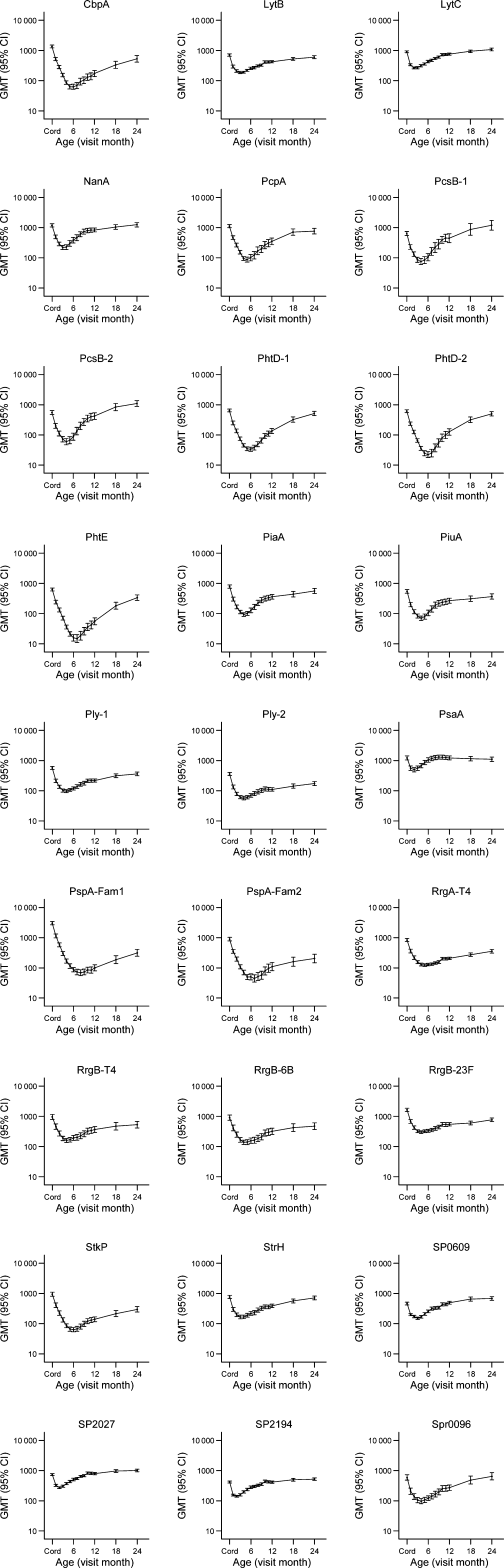
Kinetics of serum IgG antibodies to 27 pneumococcal protein antigens in infants. The graphs show monthly antibody data for 222 infants from birth to 24 months of age (GMTs with 95% confidence intervals).

In 222 infants studied, 40.3% were colonized by pneumococci at 1 month and 66.4–88.0% were colonized at each subsequent visit. One infant was not colonized by 24 months of age. Of the 3205 pneumococci cultured from these infants, 1786 (55.7%) were PCV13 serotypes, 1084 (33.8%) were non-vaccine serotypes and 335 (10.5%) were non-typeable. Geometric mean serum IgG antibody titres to all proteins fell rapidly after birth to a nadir at 4 months (median; IQR 3–5) (Fig.1). Subsequently, GMTs increased and this was largely in response to pneumococcal exposure (Figure S1). Ratios of GMT at birth (cord blood) to 12 months of age were 0.03–1.08. Twelve-month GMTs had reached cord blood levels (ratio ≥1.0) for PsaA, SP0609, SP2027 and SP2194. GMT ratios for cord and 24-month specimens were 0.10–2.00. The GMT ratio was>1.0 for seven proteins (LytC, NanA, PcsB (PcsB-1/2), SP0609, SP2027, SP2194 and Spr0096) and <0.5 for another seven (CbpA, PhtE, Ply-2, PspA (PspA-Fam1/Fam2), RrgA-T4, RrgB-23F and StkP). Evidence of increases in specific IgG in the absence of detectable colonization was seen for certain proteins, including pneumolysin (Figure S1).

### Antibody responses to pneumococcal acquisitions in infants

Thirty-six of the infants had complete 24-month swab/serum collections: median age at pneumococcal acquisition was 45.5 days; all 36 infants had been colonized by 7 months (Figure S2). Pneumococci were detected in 20 swabs per infant (median; IQR 16–21). Infants were colonized by seven individual serotypes (median; IQR 6–8). One hundred and six out of 305 (34.8%) pneumococcal nasopharyngeal acquisitions were of serotypes 6B, 14, 19F, 19A and 23F: 35/36 infants (97.2%) carried at least one of these serotypes and 23/36 (63.8%) carried ≥2. Serotype 19A was acquired later than the other serotypes (p 0.03; Figure S3).

**fig 2:**
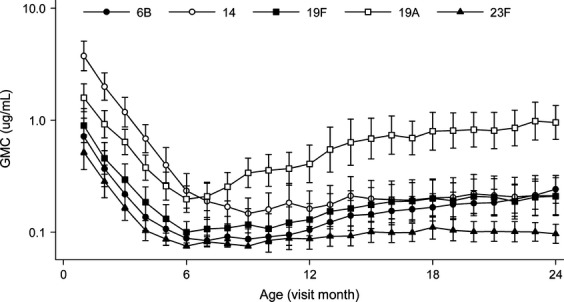
Kinetics of serum IgG antibodies to five pneumococcal capsular polysaccharides. The graph shows monthly antibody data for 36 infants from 1 to 24 months of age (GMCs with 95% confidence intervals).

Serum anti-capsular IgG were highest in the first infant sample, reflecting maternally-derived antibodies. Antibody concentrations declined until 6 months of age, with significant variation between serotypes (p 0.02) (Fig.2, Table2). Following this nadir, IgG concentrations increased modestly: none of the geometric mean concentrations (GMC) at 24 months were equal to the 1-month values (Table2). Between 6 and 24 months of age, the greatest increase was seen in anti-19A antibodies (mean fold-change, 4.91; 95% CI, 3.31–7.28) and the smallest in anti-14 antibodies (mean fold-change, 0.91; 95% CI, 0.54–1.52) (p <0.001). At 24 months, measurable anti-capsular IgG antibodies were found in the sera of 19.4–97.1% of infants, varying by serotype (Table2). Serum anti-capsular IgG concentration of ≥0.35 mg/L may protect against invasive disease caused by the homologous serotype 18,19. By 24 months of age the proportion of infants with anti-capsular IgG concentrations above this threshold varied considerably by serotype: 5.6% (95% CI, 0.1–18.7) for 23F, 30.6% (95% CI, 16.3–48.1) for 19F, 33.3% (95% CI, 18.6–60.0) for 6B, 38.9% (95% CI, 23.1–56.5) for 14, and 85.7% (95% CI, 67.2–93.6) for 19A (p <0.001). Serum anti-capsular IgG concentrations of ≥5.0 mg/L may be required to protect against nasopharyngeal colonization 4. By 24 months this concentration was only achieved by three infants (8.3%) for a single serotype, 19A.

**Table 2:** Geometric mean serum anti-capsular IgG antibody concentrations in 36 infants sampled over the first 2 years of life

Serotype	GMC (95% CI), mg/L					Infants with detectable IgG at 24 months, *N* (%)
	1 month	6 months	12 months	18 months	24 months	
6B	0.71 (0.48–1.04)	0.09 (0.07–0.10)	0.11 (0.08–0.13)	0.17 (0.13–0.22)	0.24 (0.18–0.32)	28/36 (77.8)
14	3.74 (275–5.08)	0.23 (0.16–0.33)	0.16 (0.11–0.23)	0.20 (0.14–0.30)	0.21 (0.14–0.32)	18/36 (50.0)
19F	0.90 (0.63–1.27)	0.10 (0.08–0.12)	0.13 (0.09–0.18)	0.20 (0.14–0.29)	0.21 (0.14–0.31)	16/36 (55.6)
19A	1.58 (1.19–2.11)	0.20 (0.15–0.26)	0.41 (0.28–0.60)	0.80 (0.54–1.17)	0.95 (0.67–1.35)	34/35 (97.1)
23F	0.52 (0.36–0.74)	0.08 (0.08–0.08)	0.09 (0.07–0.11)	0.11 (0.09–0.14)	0.10 (0.08–0.12)	7/36 (19.4)

Detectable IgG was defined as an anti-capsular IgG concentration of ≥0.15 mg/L.

Serum specimens taken at 1 month of age were used to assess the contribution of maternally-derived antibodies to the risk/timing of nasopharyngeal acquisition by the homologous serotype over the first 2 years of life: no significant associations were detected (Table3). Anti-protein antibody kinetics in these infants were similar to the larger cohort (Figure S4).

**Table 3:** Assessment of the effect of maternally derived serum anti-capsular IgG antibodies on the risk and timing of nasopharyngeal acquisition of the homologous serotype in the infant over the first 24 months of life

Serotype	Risk of acquisition OR (95% CI)	p-Value	Timing of acquisition HR (95% CI)	p-Value
6B	1.06 (0.23–4.91)	0.9	0.99 (0.32–3.11)	1.0
14	0.54 (0.07–3.85)	0.5	0.65 (0.14–3.00)	0.6
19F	0.64 (0.11–3.63)	0.6	0.77 (0.32–1.81)	0.5
19A	9.86 (0.46–213.16)	0.1	6.49 (0.46–91.35)	0.2
23F	1.91 (0.37–9.75)	0.4	1.41 (0.46–4.34)	0.5

Logged serum anti-capsular IgG concentrations in the infant specimen collected at 1 month of age were compared with homologous serotype acquisition in univariate logistic regression and Cox proportional hazards models. An OR of <1 indicated a lower risk of nasopharyngeal acquisition with increasing concentrations of anti-capsular IgG. An HR of <1 indicated a longer interval to acquisition with increasing concentrations of anti-capsular IgG.

In a univariate analysis, the proportion of pneumococcal acquisitions resulting in a ≥2-fold homologous anti-capsular IgG response varied by serotype: 11.1% (2/18) for 6B, 15.4% (4/26) for 23F, 23.1% (3/13) for 14, 25.0% (9/36) for 19F, and 50.0% (4/8) for 19A. Controlling for age and previous homologous serotype exposure, nasopharyngeal acquisition of serotypes 19A, 23F, 14 or 19F was associated significantly with a ≥2-fold antibody response to the homologous capsule (OR, 12.84, 7.52, 6.52, 5.33; p <0.05) (Table4). This was not the case for acquisitions of serotype 6B (p 0.3). Acquisition of a heterologous serotype was not associated with a ≥2-fold rise in anti-capsular antibodies to these serotypes (Table4). In contrast, acquisition of pneumococcal serotypes in the nasopharynx was not significantly associated with a ≥2-fold rise in antibodies to any of the protein antigens (Table5). The proportion of pneumococcal acquisitions associated with a ≥2-fold antibody response ranged from 3.1% (RrgB-23F) to 12.9% (SP0609) (data not shown).

**Table 4:** Effect of pneumococcal colonization events on serum IgG anti-capsular antibody responses in 36 infants sampled over the first 2 years of life

Capsular antibody	Homologous colonization, OR (95% CI)	p-Value	Heterologous colonization, OR (95% CI)	p-Value
6B	2.48 (0.53–11.68)	0.3	1.43 (0.78–2.60)	0.2
14	6.52 (1.73–24.54)	0.006	0.53 (0.24–1.17)	0.1
19F	5.33 (2.35–12.10)	<0.001	1.19 (0.67–2.11)	0.6
19A	12.84 (3.01–54.82)	0.001	1.28 (0.77–2.14)	0.3
23F	7.52 (2.21–25.60)	0.001	1.17 (0.45–3.01)	0.7

The results are summarized as the odds ratio (95% CI) for a ≥2-fold rise in antibody concentration in response to acquisition of pneumococci in the nasopharynx. Heterologous serotype acquisition was defined as the acquisition of any other pneumococcal type, including non-typeable strains. Age and previous carriage of the homologous serotype were adjusted for in the model.

**Table 5:** Effect of pneumococcal colonization events on serum IgG anti-protein antibody responses in 36 infants sampled over the first 2 years of life

Protein antibody	Colonization, OR (95% CI)	p-Value
CbpA	1.04 (0.66–1.65)	0.9
LytB	0.92 (0.44–1.92)	0.8
LytC	0.79 (0.39–1.58)	0.5
NanA	1.20 (0.71–2.01)	0.5
PcpA	1.01 (0.66–1.56)	1.0
PcsB-1	0.71 (0.44–1.14)	0.2
PcsB-2	0.64 (0.39–1.05)	0.08
PhtD-1	1.41 (0.84–2.35)	0.2
PhtD-2	1.24 (0.78–2.00)	0.9
PhtE	1.25 (0.78–2.00)	0.4
PiaA	1.05 (0.64–1.70)	0.9
PiuA	1.33 (0.81–2.18)	0.3
Ply-1	1.46 (0.78–2.76)	0.2
Ply-2	1.48 (0.80–2.76)	0.2
PsaA	1.34 (0.76–2.39)	0.3
PspA-Fam1	1.30 (0.84–2.04)	0.2
PspA-Fam2	1.18 (0.75–1.84)	0.5
RrgA-T4	1.23 (0.61–2.45)	0.6
RrgB-T4	0.73 (0.42–1.28)	0.3
RrgB-6B	0.76 (0.45–1.29)	0.3
RrgB-23F	0.62 (0.29–1.34)	0.2
StkP	1.06 (0.59–1.90)	0.8
StrH	1.46 (0.84–2.54)	0.2
SP0609	1.23 (0.79–1.93)	0.4
SP2027	0.81 (0.43–1.54)	0.5
SP2194	0.64 (0.32–1.31)	0.2
Spr0096	0.81 (0.47–1.39)	0.5

The results are summarized as the age-adjusted odds ratios (95% CI) for a ≥2-fold rise in antibody titre in response to acquisition of pneumococci in the nasopharynx.

## Discussion

This manuscript describes the development of serum IgG to a large panel of pneumococcal surface/virulence proteins, and the effect of transplacental transfer of these antibodies, in a population of SE Asian infants where pneumococcal colonization occurs early in life. Comparison of anti-protein antibody with anti-capsular antibody responses was made.

Maternally-derived serum IgG antibodies to the pneumococcal proteins studied did not appear to protect infants from early pneumococcal colonization. In a multivariate model, only environmental factors significantly influenced the timing of infant colonization. Studies from the Philippines and Papua New Guinea concluded that maternally derived anti-pneumolysin antibodies were associated with delayed infant colonization 20,21. Reproducing the logistic regression analysis described in the Philippines study did demonstrate a trend towards delayed colonization with higher anti-Ply titres in our cohort (data not shown), but the Cox model remained a more appropriate analysis for the current dataset. Differences in environmental factors, nasopharyngeal sampling frequency or assay/statistical methodology may account for the differences between studies.

We found no evidence that maternally-derived anti-capsular antibodies delayed or prevented colonization in infants. None of the 1 month of age anti-capsular IgG GMCs were close to the putative protective threshold of 5 mg/L 4. Anti-capsular IgG concentrations ≥5 mg/L were only achieved in three infants by 24 months, also suggesting that serum anti-capsular IgG is unlikely to protect against colonization in non-PCV immunized young children 22.

The absence of a protective effect of antibody on colonization may reflect the likely high level of exposure to pneumococci in the community in this population but has also been documented in European studies 4,23,24. These findings were also in agreement with a murine study, which concluded that serum antibodies to PsaA, PspA or PpmA were not protective against nasopharyngeal colonization 25. However, these results do not necessarily predict the protective quality of the immune response elicited by active immunization against any of the antigens.

Infants began developing serum antibodies to selected proteins from 2 months of age and to capsular polysaccharides from 6 months of age, largely in response to nasopharyngeal colonization. Pneumococcal acquisitions were more likely to result in a clear homologous anti-capsular IgG response rather than anti-protein responses. However, certain proteins were clearly more immunogenic than others. Antibodies to PsaA rose rapidly from 2 months and the GMT at 12 months was greater than the cord blood GMT, in agreement with previous studies 20,26. A small number of proteins were poorly immunogenic throughout the study period. Although antibodies to pneumococcal pilus proteins (RrgA, B and C) are highly protective against invasive disease in mice 27, antibody responses to pilus following colonization were relatively modest, probably reflecting the non-conserved nature of the pilus-encoding genes amongst pneumococci 28. We previously demonstrated a PI-1 prevalence of 35.2% in carried pneumococci from this cohort 29.

At 24 months a proportion of infants had serum anti-capsular IgG concentrations above the putative protective threshold for invasive disease (0.35 mg/L), from 5.6% (23F) to 85.7% (19A). These data support the hypothesis that, although anti-capsular antibodies are an important defence against invasive pneumococcal infection, other immune mechanisms, perhaps mucosal antibody produced by mucosal B cells or T cells, are also critical for the observed decline in incidence of invasive disease caused by all common serotypes after the first year of life 30.

The early acquisition and persistence of pneumococcal colonization in the nasopharynx of the study infants were both important positive and negative aspects of our study. After 3 months of age>2/3 of infants were colonized at each time-point, permitting determination of the kinetics of serum IgG development to pneumococcal antigens in a population almost continually exposed to the organism. In terms of pneumococcal colonization, this population is similar to many developing world settings where effective pneumococcal vaccines with broad serotype coverage will have the most benefit. However, this high level of colonization, coupled with the monthly swabbing interval, limited the ability to detect small differences in the timing of first pneumococcal acquisition in relation to cord blood antibody titres. In Papua New Guinea weekly swabs were taken from infants during the first month of life, resulting in a higher resolution than we achieved 21.

In conclusion, our results demonstrate a considerable variation in the immunogenicity of pneumococcal surface/virulence proteins in infancy. Acquisition of pneumococci in the nasopharynx resulted in demonstrable anti-capsular, but not anti-protein, responses. Evidence of antibody-mediated protection against colonization was not identified. Further work on the immunological mechanisms underlying protection from nasopharyngeal acquisition is required.

## Supporting Information

Table S1 Serum specimens analysed in the study. **Table S2.** Univariate and multivariate analyses of the effect of cord blood anti-protein titres on timing of first pneumococcal colonization in infants. **Figure S1.** Geometric mean serum IgG antibody titres to pneumococcal proteins by age. **Figure S2.** Cumulative proportion of infants colonized by (i) all pneumococci (grey line) and (ii) target pneumococcal serotypes (coloured lines) by age. **Figure S3.** Age at first acquisition of target pneumococcal serotypes. **Figure S4.** Geometric mean serum IgG antibody titres to pneumococcal proteins by age.
