# Supplementary material for: Serum antibody responses to pneumococcal colonization in the first 2 years of life: results from an SE Asian longitudinal cohort study
Source: Clin Microbiol Infect. 2013 Jul 5;19(12):E551–8. doi: 10.1111/1469-0691.12286 (PMC4282116; doi:10.1111/1469-0691.12286)

Supporting information

Serum antibody responses to pneumococcal colonisation in the first two years of life: results from a SE Asian longitudinal cohort study

Paul Turner, Claudia Turner, Nicola Green, Lindsey Ashton, Eh Lwe, Auscharee Jankhot, Nicholas P Day, Nicholas J White, François Nosten, and David Goldblatt

Table S1. Serum specimens analysed in the study.

**Time point (Age, m)**		**Number of sera**		**Time point (Age, m)**		**Number of sera**	
		**Protein Ab only**	**Capsular & Protein Ab**			**Protein Ab only**	**Capsular & Protein Ab**
**Mother**	Delivery	230	**0**	**Infant**	12	113	36
**Cord**	Delivery	184	0		13	0	36
**Infant**	1	98	36		14	0	36
	2	98	36		15	0	36
	3	98	36		16	0	36
	4	97	36		17	0	36
	5	97	36		18	63	36
	6	114	36		19	0	36
	7	97	36		20	0	36
	8	96	36		21	0	36
	9	98	36		22	0	36
	10	96	36		23	0	36
	11	98	36		24	83	36

Table S2. Univariate and multivariate analyses of the effect of cord blood anti-protein titres on timing of first pneumococcal colonisation in infants.

Results from 179 infants were included in these analyses. Only those antibodies with a univariate p-value of <0.05 are shown for clarity. These antibodies, and all environmental/household variables, were included in the multivariate analysis. HR >1 indicate earlier colonisation and HR<1 indicate delayed colonisation.

**Factor**	**Univariate model**		**Multivariate model**	
	**Hazard Ratio (95% CI)**	**p-value**	**Hazard Ratio (95% CI)**	**p-value**
Antibodies against pneumococcal surface / virulence proteins				
Anti-PiuA IgG (log10 titre)	0.68 (0.47 – 0.98)	0.04	0.70 (0.45 – 1.08)	0.1
Anti-Spr0096 IgG (log10 titre)	0.74 (0.57 – 0.95)	0.02	0.82 (0.62 – 1.09)	0.2
Environmental / household factors				
Household size >5 people	1.36 (1.00 – 1.86)	0.05	1.21 (0.86 – 1.70)	0.3
Children <5y in the house	1.46 (1.08 – 1.99)	0.01	1.39 (0.98 – 1.96)	0.06
Mother smoker	1.58 (1.10 – 2.28)	0.01	1.49 (1.01 – 2.21)	0.046
Ethnic group:				
Sgaw Karen	-	-	-	-
Pwo Karen	1.03 (0.62 – 1.69)	0.9	0.93 (0.54 – 1.59)	0.8
Muslim	1.17 (0.74 – 1.88)	0.5	1.31 (0.78 – 2.20)	0.3
Other	1.49 (0.61 – 3.67)	0.4	1.75 (0.69 – 4.48)	0.2
Season of birth:				
Hot (March – May)	-	-	-	-
Wet (June – October)	0.62 (0.42 – 0.94)	0.02	0.62 (0.40 – 0.95)	0.03
Cool (November – February)	1.00 (0.66 – 1.50)	1.0	1.16 (0.75 – 1.79)	0.5
Home delivery	2.39 (1.25 – 4.57)	0.008	1.74 (0.88 – 3.44)	0.1
Prematurity	0.62 (0.34 – 1.52)	0.1	0.74 (0.29 – 1.91)	0.5
Mother colonised at birth	1.54 (1.06 – 2.24)	0.03	1.50 (1.00 – 2.25)	0.05
Antibiotics in the neonatal period	0.85 (0.54 – 1.33)	0.5	1.32 (0.78 – 2.25)	0.3

Figure S1. Geometric mean serum IgG antibody titres to pneumococcal proteins by age. Solid lines represent infants who had become colonised by *S. pneumoniae* and dashed lines represent infants remaining uncolonised. Age "0" indicates cord blood specimens. Data from all 222 infants included in the anti-protein antibody analyses.

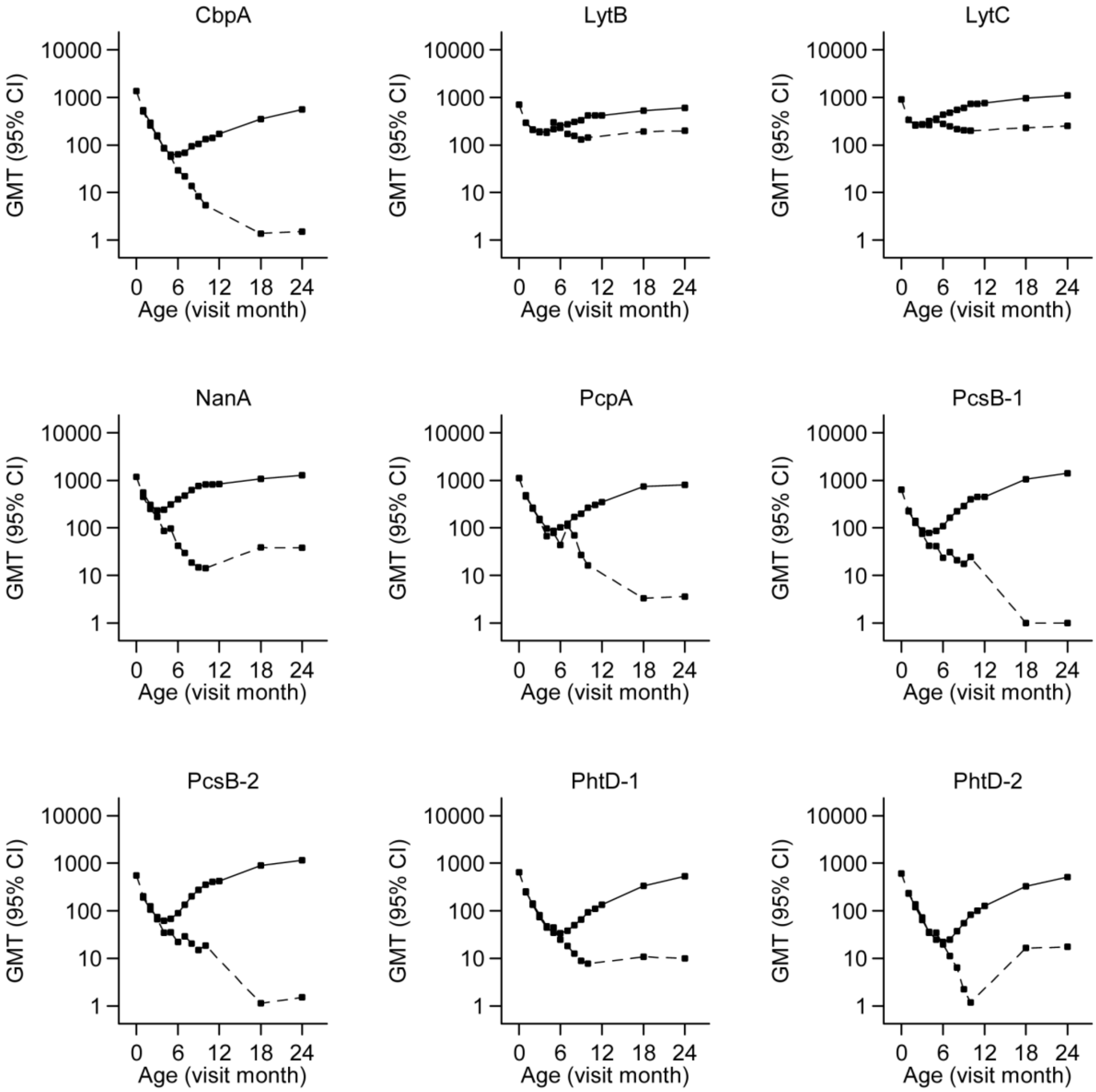

Figure S1 cont.

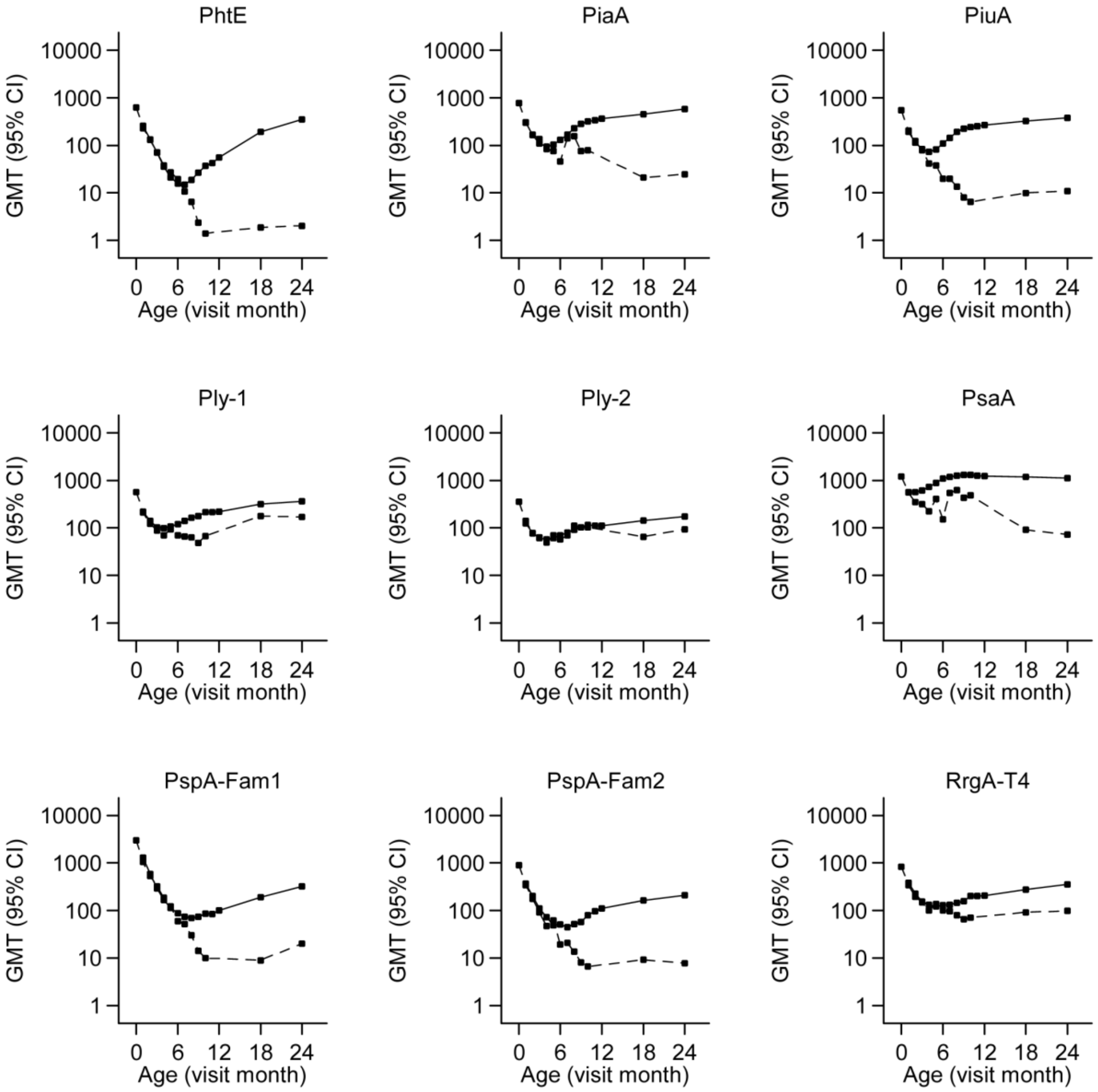

Figure S1 cont.

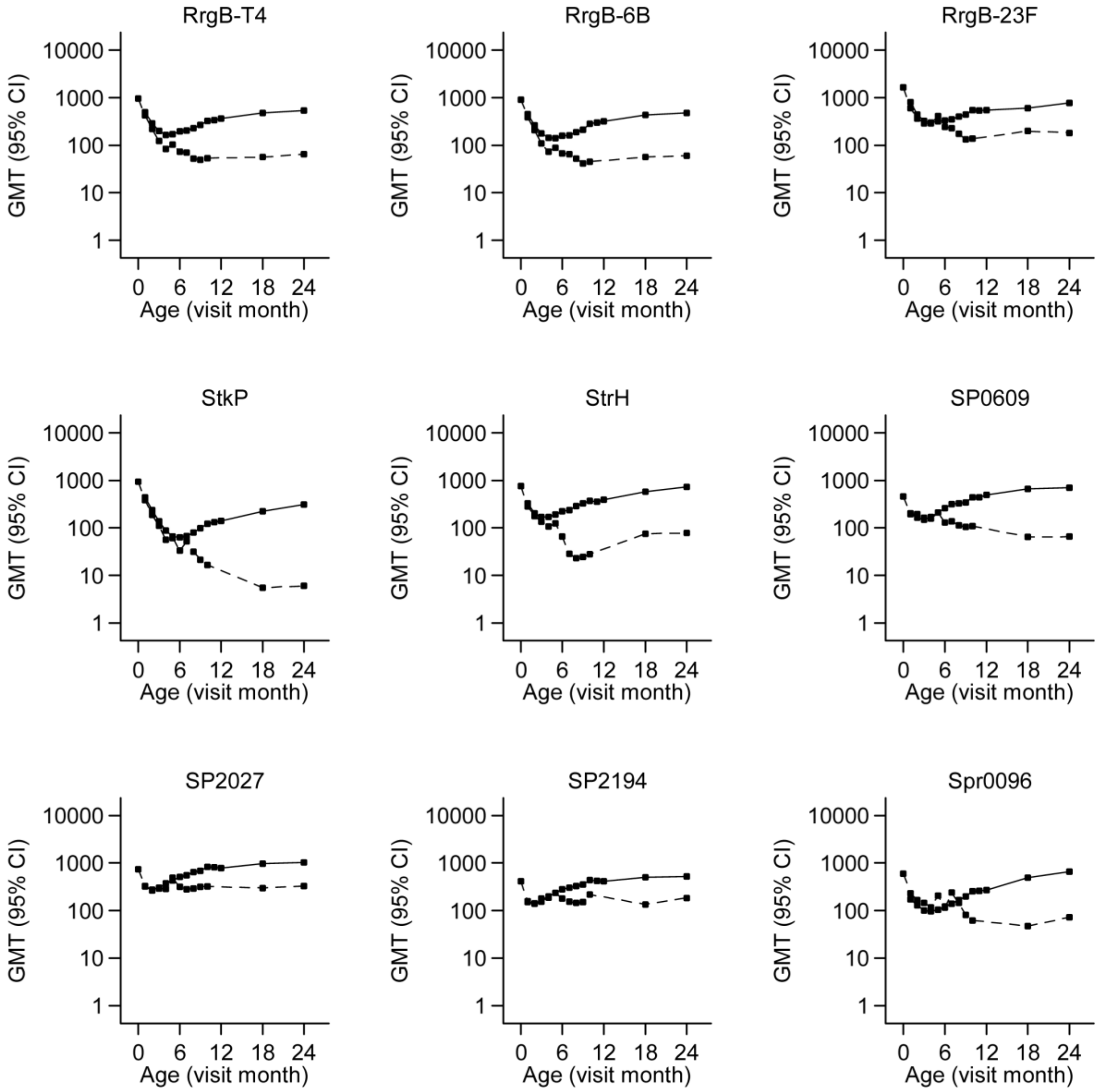

Figure S2. Cumulative proportion of infants colonised by (i) all pneumococci (grey line) and (ii) target pneumococcal serotypes (coloured lines) by age.
Data from 36 infants included in the combined anti-capsular and anti-protein antibody analyses.

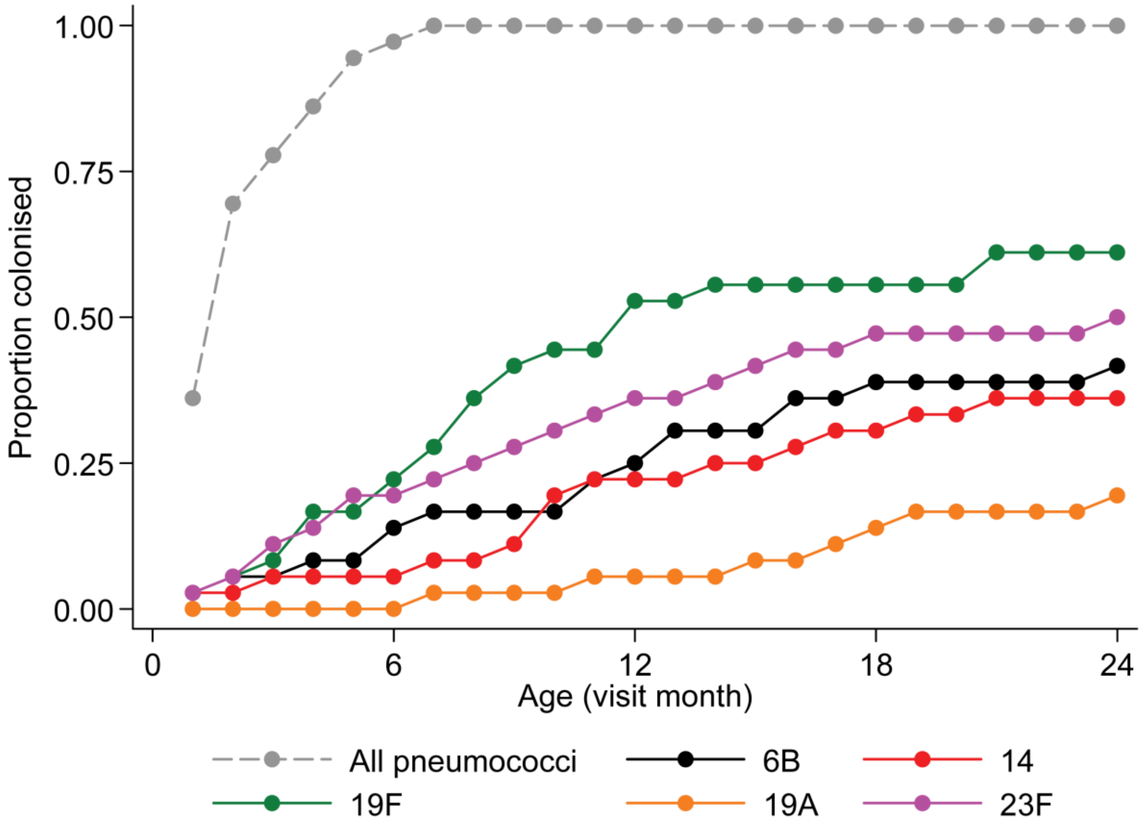

Figure S3. Age at first acquisition of target pneumococcal serotypes.
Acquisition was defined as the first monthly visit in which the serotype was detected in an infant's nasopharyngeal swab. Data from 36 infants included in the combined anti-capsular and anti-protein antibody analyses.

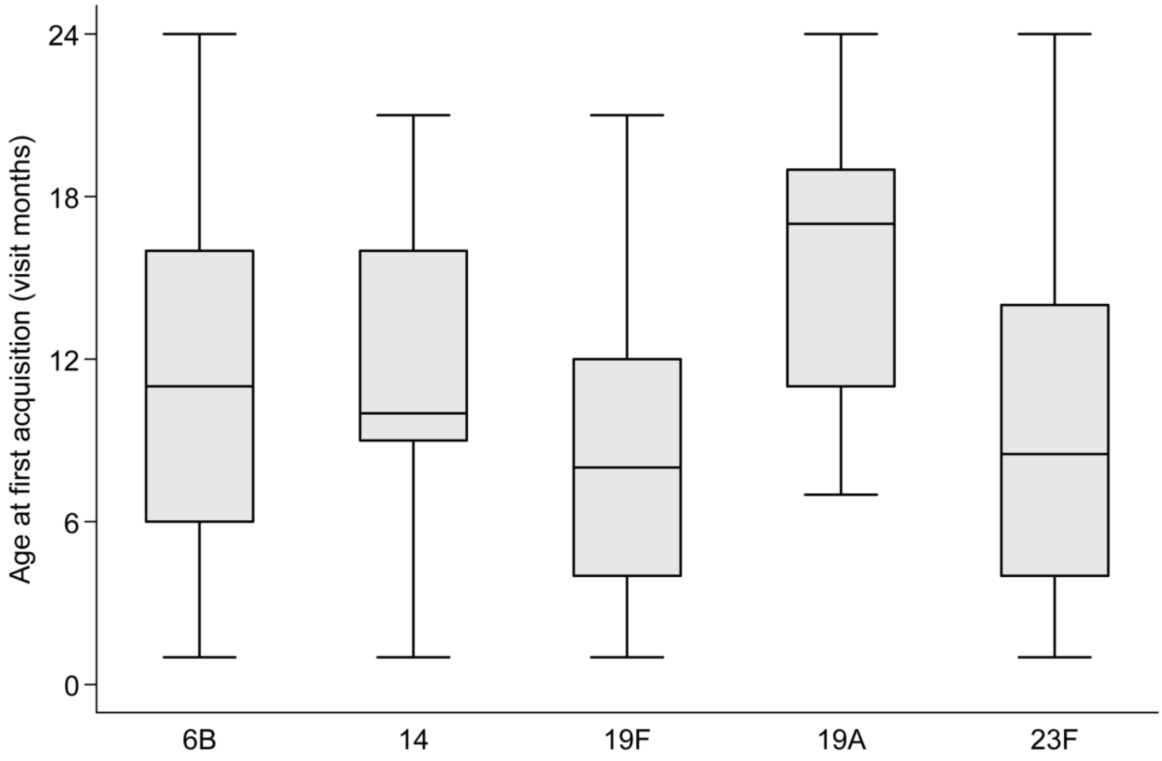

Figure S4. Geometric mean serum IgG antibody titres to pneumococcal proteins by age. Data from 36 infants included in the combined anti-capsular and anti-protein antibody analyses. For each antigen, the black line represents the antibody GMT and the grey shaded area defines its 95% CI.

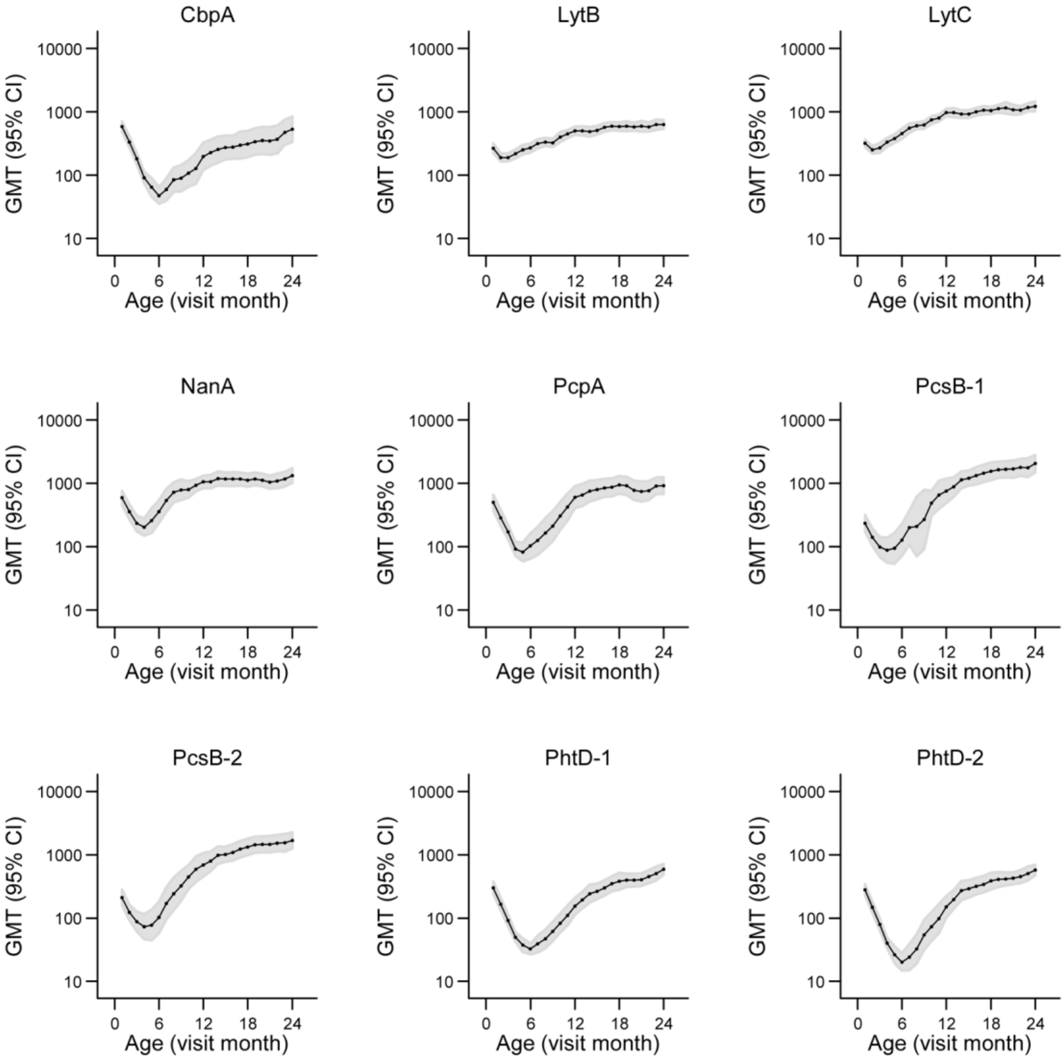

Figure S4 cont.

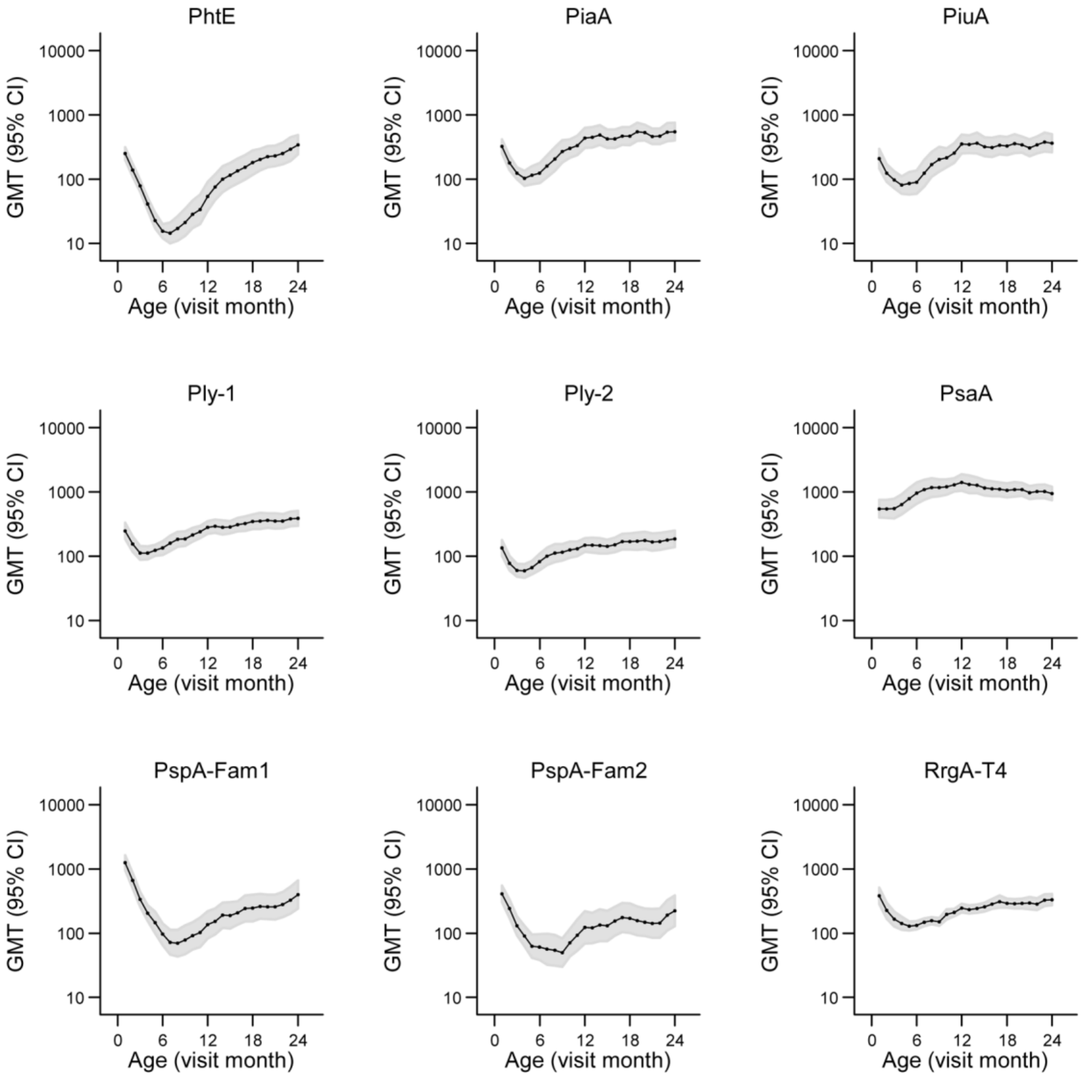

Figure S4 cont.

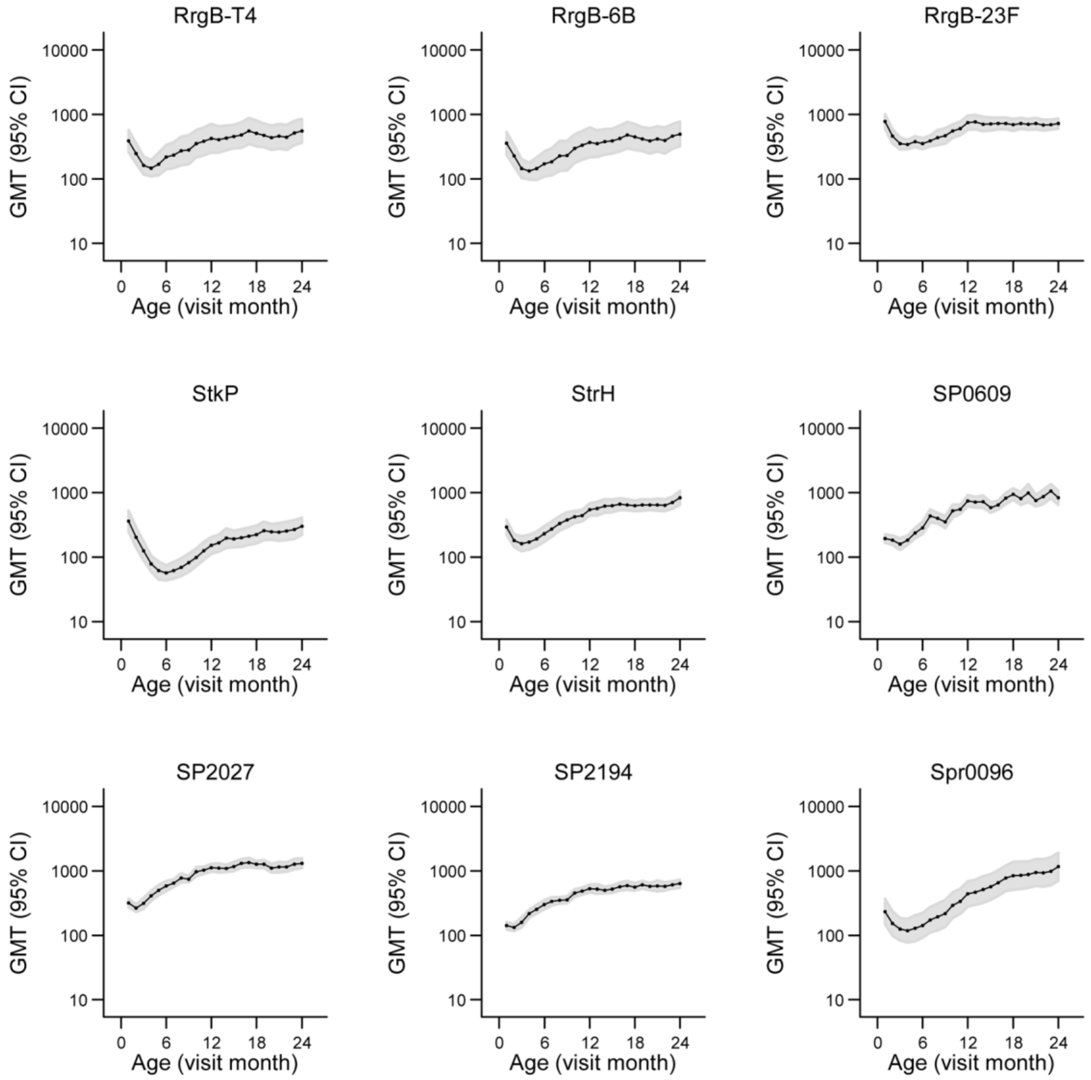

Supplement: Table S1 — Serum specimens analysed in the study. Table S2. Univariate and multivariate analyses of the effect of cord blood anti-protein titres on timing of first pneumococcal colonization in infants. Figure S1. Geometric mean serum IgG antibody titres to pneumococcal proteins by age. Figure S2. Cumulative proportion of infants colonized by (i) all pneumococci (grey line) and (ii) target pneumococcal serotypes (coloured lines) by age. Figure S3. Age at first acquisition of target pneumococcal serotypes. Figure S4. Geometric mean serum IgG antibody titres to pneumococcal proteins by age. [file clm0019-E551-SD1.pdf]
